# Supplementary material for: The Spectrum of Clinical and Serological Features of COVID-19 in Urban Hemodialysis Patients
Source: J Clin Med. 2020 Jul 16;9(7):2264. doi: 10.3390/jcm9072264 (PMC7408812; doi:10.3390/jcm9072264)
Supplement: Supplementary file 1 [file jcm-09-02264-s001.pdf]

Supplementary Materials

Table S1. Characteristics of polymerase chain reaction (PCR) negative patients, according to diagnosis of COVID-19. Only symptoms presented by at least 1 patient are represented. Data presented as mean±SD, median (interquartile range) or counts (percentage).

**Characteristics**	**Positive Serology (*n* = 8)**		Negative Serology, Clinical Diagnosis (*n* = 2)		No COVID-19 (*n* = 18)	
Age, years	69.125	± 17.1	71	±7.07	63.3	±13.41
Dialysis center, n (%)						
FJD	7	(87.5)	2	(100)	18	(100)
FRCSE	1	(12.5)	0		0	
Dialysis vintage, days	1027.2	±876.1	938.5		610.8	±775.5
Female, n (%)	4	(50)	0		4	(22)
Hospital admission, n (%)	1	(12.5)	2	(100)	3	(16.7)
Symptoms, n (%)						
Asymptomatic	6	(75)	0		14	(77.8)
Dyspnea	0		0		1	(5.6)
Abdominal pain	0		0		1	(5.6)
Fever	1	(12.5)	2	(100)	2	(11.1)
General malaise	1	(12.5)	0		0	
Oxygen saturation, %	95%	*	96	*	96.3	±4.99
Chest X-ray, n (%)						
Normal	1	(12.5)	0		5	(27.8)
Unilateral pneumonia	0		1	(50)	1	(5.6)
Bilateral pneumonia	1	(12.5)	1	(50)	1	(5.6)
No	6	(75)	0		11	(61.1)
Clinical analysis						
Hemoglobin, g/dL	11.2	±1.46	11.95	±2.47	11.2	±1.92
Lymphocytes[n],/μL	1150	(900-1400)	400	(400-400)	1000	(850-1425)
D dimer[n], μg/L	1148.5	(701-1562)	2812	(2382-3242)	1162	(739-2160)
Ferritin, ng/mL	513	(204-1067)	482	(291-673)	423	(340-598)
Troponin I[n], ng/mL	0.04	(0.007-0.06)	0.006	(0.003-0.008)	0.05	(0.021-0.07)
IL6[n], pg/mL	8.94	(3.27-18.26)	31.2	(25.95-36.45)	10.34	(7.45-15.07)
Serology, n (%)						
IgM, n (%)						
Positive	8	(100)	0			
Negative	0		2	(100)	18	(100)
IgG, n (%)						
Positive	4	(50)	0			
Negative	4	(50)	2	(100)	18	(100)
IgG + IgM positive, n (%)	4	(50)	0		0	

* data for *n* = 2. [n] Normal range: Lymphocytes 1200 – 5000/ μl; D-dimer 68–494 μg/l, Troponin I < 0.08 ng/mL, IL6 < 7 pg/mL.

Table S2. Characteristics of patients with positive and negative PCR. Data presented as mean±SD, median (interquartile range) or counts (percentage).

**Characteristics**	**Positive PCR (*N* = 38)**		**Negative PCR (*N* = 10)**		**P value**
Initial dialysis center, *n* (%)					
FJD	13	(34.2)	10	(100)	
FRCSE	25	(65.8)	0		
Symptoms, *n* (%)					0.001
Asymptomatic	5	(13.2)	6	(60)	
Asthenia	1	(2.6)	0		
Diarrhea	4	(10.5)	0		
Dyspnea	4	(10.5)	0		
Abdominal pain	1	(2.6)	0		
Fever	12	(31.5)	3	(30)	
Cough	4	(10.5)	0		
Chest pain	1	(2.6)	0		
General malaise	4	(10.5)	1	(10)	
Nausea	1	(2.6)	0		
Other manifestations					
AV blockade	1	(2.6)	0		

Table S3. Analysis of PCR positive patients, according to hospitalization related to disease severity. Data presented as mean ± SD, median (interquartile range) or counts (percentage).

Characteristics	Hospitalization				*P* value
	No (*N* = 17)		Yes (*N* = 21)		
Initial dialysis center, *n* (%)					
FJD	6	(37.5)	7	(31.2)	
FRCSE	10	(62.5)	15	(68.2)	
Symptoms, *n* (%)					ns
Asymptomatic	5	(31.25)	0		
Asthenia	1	(6.25)	0		
Diarrhea	2	(12.5)	2	(9.1)	
Dyspnea	2	(12.5)	2	(9.1)	
Abdominal pain	1	(6.25)	0		
Fever	4	(25)	8	(36.3)	
Cough	1	(6.25)	3	(13.6)	
Chest pain	0		1	(4.5)	
General malaise	0		4	(18.2)	
Nausea	0		1	(4.5)	
Other manifestations					
AV blockade	0		1	(4.5)	

Table S4. Analysis of PCR negative patients, according to hospitalization related to disease severity. Data presented as mean ± SD, median (interquartile range) or counts (percentage).

Characteristics	Hospitalization				*P* value
	No (*N* = 7)		Yes (*N* = 3)		
Initial dialysis center, *n* (%)					
FJD	7	(100)	2	(66.7)	
FRCSE	0		1	(33.3)	
Symptoms, *n* (%)	1	(14.3)	3	(100)	
Asymptomatic	6	(85.7)	0		
Asthenia	0		0		
Diarrhea	0		0	(80)	
Dyspnea	0		0		
Abdominal pain	0		0		
Fever	1	(14.7)	2	(66.7)	
Cough	0		0		
Nausea	0		0		

Table S5. COVID-19 hemodialysis cohorts.

Series (Reference)	Total patients in unit	Total patients tested *n* (%)	Testing criteria	PCR+ *n* (%) ***	Clinical or serological diagnosis n (%)***	COVID-19					
						**Overall incidence********	**Asymptomatic *n* (%)**	**Mortality (PCR+)**	**Mortality (all)**	**Age (all)**	**Age (deceased)**
Spain (A+B)	290	156 (54%)	Symptomatic firs, close contacts, screening	74/156 (47%)	11/156 (7%)	85/290 (29%)	26/75 (35%)	11/75 (15%)	11/85 (13%)-	-	-
A[36]	90	90 (100%)	Screening	36/90 (40%)	1/90 (1%)	37/90 (41%)	15/37 (40%)	6/36 (17%)	6/37 (16%)	ND	80±15
B (Present work)	200	66 (33%)	Symptomatic, close contacts. Screening*	38/66 (58%)	10/66 (15.2%)	48/200 (24%)	11/38 (29%)	5/38 (13%)	5/48 (10%)	73±13	79± 4
Italy (C+D)	657	ND	ND	62/657 (9%)	ND	62/657 (9%)	ND	23/62 (37%)	23/62 (37%)	-	ND
C[37]	400	ND	ND	21/400 (5%)	ND	21/400 (5%)	ND	5/21 (24%)	5/21 (24%)	ND	ND
D[38]	257	ND	ND	41/257 (16%)	ND	41/257 (16%)	ND	18/41 (41%)	18/41 (41%)	73±11	ND
China (E+F)	857	857 (100%)	Screening	103(12%)	ND	103 (12%)	50/103 (48%)	18/103 (17%)	18/103 (17%)	-	-
E[17]	230	230 (100%)	Screening	37 (16%)	ND	37 (16%)	27/37 (72%)	6/37 (16%)	6/37 (16%)	66 (55-81)	ND
F[30]	627	627 (100%)	Screening	66 (10%)	ND	66 (10%)	23/66 (26%)	12/66 (18%)	12/66 (18%)	61±13	ND

ND: no data. * Screening for remaining no tested patients in the unit receiving all COVID-19 patients from two coordinated dialysis units. ** same as for PCR+ patients. *** out of tested patients. **** out of the whole dialysis unit/s.
